# L19-Conjugated Gold Nanoparticles for the Specific Targeting of EDB-Containing Fibronectin in Neuroblastoma

**DOI:** 10.3390/pharmaceutics18010024

**Published:** 2025-12-24

**Authors:** Chiara Barisione, Silvia Ortona, Veronica Bensa, Caterina Ivaldo, Eleonora Ciampi, Simonetta Astigiano, Michele Cilli, Luciano Zardi, Mirco Ponzoni, Domenico Palombo, Giovanni Pratesi, Pier Francesco Ferrari, Fabio Pastorino

**Affiliations:** 1 Department of Surgical and Integrated Diagnostic Sciences, University of Genoa, 16132 Genoa, Italy; 2 IRCCS Ospedale Policlinico San Martino, 16132 Genoa, Italy; 3 Department of Experimental Medicine, University of Genoa, 16132 Genoa, Italy; 4 Laboratory of Experimental Therapies in Oncology, IRCCS Istituto Giannina Gaslini, 16147 Genoa, Italy; veronicabensa@gaslini.org (V.B.); mircoponzoni@gaslini.org (M.P.);; 5 Animal Facility, IRCCS Ospedale Policlinico San Martino, 16132 Genova, Italy; 6 Research Center for Biologically Inspired Engineering in Vascular Medicine and Longevity, University of Genoa, 16145 Genoa, Italy; 7 Clinic of Vascular and Endovascular Surgery, IRCCS Ospedale Policlinico San Martino, 16132 Genoa, Italy; 8 Department of Civil, Chemical and Environmental Engineering, University of Genoa, 16145 Genoa, Italy

**Keywords:** neuroblastoma, fibronectin extra-domain B, L19 gold nanoparticles, photoacoustic imaging

## Abstract

**Background/Objectives**: Neuroblastoma (NB) is the most common extracranial solid tumor in children and accounts for 12–15% of pediatric cancer-related deaths. Current multimodal therapies lack specific cellular targets, causing systemic toxicity and drug resistance. The development of innovative tumor-targeted nanoformulations might represent a promising approach to enhance NB diagnosis and antitumor efficacy, while decreasing off targets side effects. Fibronectin extra-domain B (FN-EDB) is upregulated in the tumor microenvironment. **Methods**: FN-EDB expression was evaluated by immunohistochemical staining in cell line-derived and tumor patient-derived animal models of NB. A gold nanoparticle, decorated with an antibody (Ab) recognizing FN-EDB (L19-AuNP) was developed by the company Nano Flow and its tumor binding was tested by ELISA in vitro and in patient-derived xenograft (PDX) models of NB by photoacoustic imaging in vivo. **Results**: All animal models of NB used have been shown to express FN-EDB. L19 Ab demonstrated excellent binding specificity to FN-EDB both when used in free form and after conjugation to AuNP. Compared to the non-functionalized (no Ab L19-coupled) AuNP, which showed an increase in PDI and zeta potential over time, making them unsuitable for use in in vivo studies, L19-AuNP demonstrated good stability. In vivo, L19-AuNP specifically homed into PDX models of NB, accumulating better in tumors expressing higher levels of FN-EDB. Negligible distribution to healthy organs occurred. **Conclusions**: In this preliminary study, L19-AuNP was shown to be a novel diagnostic tool specifically for binding NB expressing FN-EDB, paving the way for the development of theranostic nanoformulations co-encapsulating gold moiety and standard-of-care therapy for NB.

## 1. Introduction

Neuroblastoma (NB) originates from cells of the developing sympathetic nervous system and is the most common and aggressive extracranial solid tumor in childhood [1,2]. Although recent therapeutic advances have improved clinical outcomes, current multimodal treatments still lack cell-specific targets, leading to systemic toxicity and drug resistance [1]. These limitations highlight the need for innovative, tumor-targeted therapeutic strategies.

Fibronectin extra domain B (FN-EDB) is an oncofetal isoform of fibronectin generated by alternative splicing [3]. It is virtually undetectable in normal adult tissues but becomes highly expressed during pathological extracellular matrix (ECM) remodeling, including in aggressive solid tumors. This sharp differential expression makes FN-EDB a reliable tumor-associated marker. Its accumulation is a defining feature of the tumor stroma and is predominantly associated with the neovasculature of malignant lesions, while remaining consistently absent from healthy tissues [4,5,6].

The abundant expression of FN-EDB in the tumor microenvironment has direct structural and functional implications. The insertion of the ED-B exon induces conformational rearrangements that reorient adjacent FNIII modules, thereby optimizing high-affinity binding to integrins (α_5_β_1_, α_v_β_3_) and promoting integrin clustering at the cell surface [7]. These effects enhance cancer cell adhesion and migration and contribute to the assembly of a pro-tumorigenic ECM.

The strict absence of FN-EDB in healthy tissues, combined with its marked overexpression in tumors, makes it a highly attractive target for antibody-based therapeutic strategies. This selectivity is exploited by agents such as the human scFv L19, which binds FN-EDB with high specificity and can deliver therapeutic payloads—including cytokines and radionuclides—directly to tumors while sparing normal organs. The unique biology of FN-EDB continues to support its clinical translation, as highlighted in recent analyses of targeted immunotherapies and drug conjugate technologies [8,9].

Targeted nanoformulations development is a promising approach to enhance antitumor efficacy while reducing side effects [10,11,12]. In this scenario, gold nanoparticles (AuNPs) are one of the nanomaterials most widely used in bioengineering, bioimaging, and biomedical therapeutics [13,14]. The great interest in these nanodevices comes from their optical properties, which are the result of an intense surface plasmon resonance effect [15,16]. The well-defined protocols present in the literature evidence the possibility to easily obtain AuNPs conjugated with biomolecules (e.g., antibodies and DNA) to be used in different biomedical applications, including biosensors [17], drug delivery [18,19], and diagnosis [20,21].

An emerging non-invasive molecular imaging technique is represented by photoacoustics (PA), which combines the advantages of optical and ultrasound properties. Specifically, a laser beam hits a target which, upon heating, emits ultrasound waves, detected by the instrument transducer, generating two- or three-dimensional images [22]. PA offers higher spatial resolution (up to 5 μm) and greater imaging depth (up to 5–6 cm) and provides improved resolution at the anatomical, functional, metabolic, and molecular levels by exploiting the optical properties of different endogenous molecular components. Moreover, this detection capability can be significantly enhanced through the use of exogenous contrast agents [23]. Currently, the potential of PA is under intensive investigation to validate its use in preclinical research and to support its possible clinical translation for cancer diagnosis, staging, and therapy [24]. A wide variety of contrast agents exists for PA imaging, and AuNPs are among the most promising because they are safe [25,26] and because of their potential for both diagnostic and therapeutic applications [23,25,27].

In this study, using scFv L19 [28,29], we demonstrate that FN-EDB is expressed in both the cell line- and patient-derived tumor microenvironment of NB. Furthermore, AuNPs were conjugated with the L19-uteroglobin-format FN-EDB [30,31]. By using PA imaging, for the first time to our knowledge, we also preliminarily show that L19-AuNP specifically home into patient-derived xenografts models of FN-EDB-expressing NB. This successful homing positions L19-AuNPs as a promising diagnostic and, in the future, theranostic platform for NB.

## 2. Materials and Methods

### 2.1. Human Samples and Mouse Models of Neuroblastoma

All procedures were performed in accordance with the relevant guidelines and regulations and approved by the CER (Regional Committee), under the protocol 104, 14 June 2011, the protocol ANTECER_Neuroblastoma: 15 December 2016, amendment 065_16/09/2019, and the protocol EudraCT: 2022-000558-27, 15 March 2022, amendment 28 July 2025. Informed consent was obtained from each patient in accordance with the Declaration of Helsinki.

Five-week-old female athymic nude-Foxn1^nu^ *(nu/nu)* mice (Envigo, Bresso, Italy) were used. In accordance with the 3Rs policy, animal studies were reviewed and approved by the licensing and ethical committee of Ospedale Policlinico San Martino and by the Italian Ministry of Health (n. 883/2020-PR, 29 September 2020, and n. 539/2024-PR, 14 June 2024).

#### 2.1.1. Orthotopic Model

The orthotopic model was used for evaluating fibronectin extra domain B (FN-EDB) expression in the neuroblastoma (NB) tumor microenvironment. Mouse NXS2 and human *MYCN* single-copy SH-SY5Y and *MYCN*-amplified IMR-32 NB cell lines (1 × 10^6^ cells in 10 μL culture medium) were inoculated in the left adrenal gland of mice (n = 1/cell line), as described [10]. Thirty days after tumor cells injection, mice were sacrificed through CO_2_ asphyxiation, and tumors were removed, cryopreserved, and immunohistochemically (IHC) evaluated for the baseline FN-EDB expression, as reported in the Immunohistochemistry Studies paragraph.

#### 2.1.2. PDX Model

**Figure 1:**
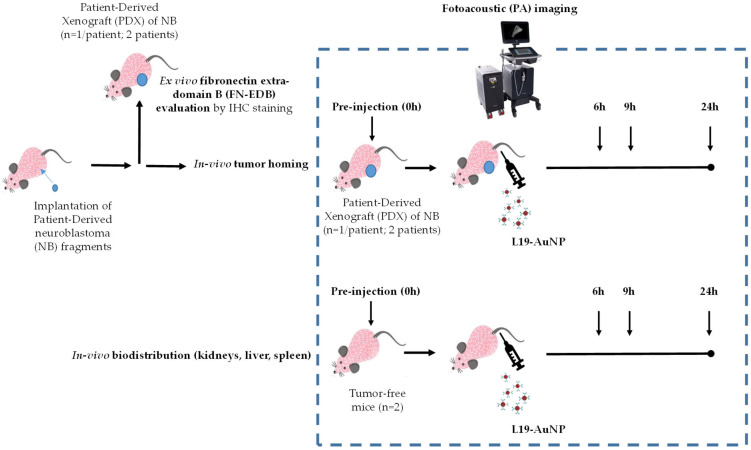
**Experimental methodology.** FN-EDB evaluation and molecular imaging of L19-AuNP on two PDX mouse models of NB and on two tumor-free mice.

The PDX model was implemented for both evaluating FN-EDB expression in the tumor microenvironment of NB and studying the tumor homing of FN-EDB-recognizing gold nanoparticles (L19-AuNP) (Figure 1).

Briefly, low-passage tumor fragments, stored in our repository [32] and derived from two patients, one with *MYCN* single-copy status (pt#1) and the other with *MYCN*-amplified status (pt#2), both affected by high-risk NB, were subcutaneously implanted in the left flank of mice (n = 1/patient). Tumors reaching an average volume of 0.8 cm^3^, following caliper measurement, were removed, cryopreserved, and immunohistochemically evaluated for the baseline FN-EDB expression, as reported above.

In the tumor homing studies, 100 μL of an L19-AuNP suspension with a final optical density of 24.3 was administered intravenously in PDX-bearing mice. The biodistribution (BD) profile study of L19-AuNP, administered as reported above, was conducted in tumor-free mice (n = 2) (Figure 1).

### 2.2. Production and Validation of the L19 Antibody

L19-uteroglobin was purified from the conditioned medium of a stable recombinant HEK293 cell line by EDB-sepharose affinity chromatography, as previously described [30]. It was shipped to the company Nano Flow (Ougree, Belgium) for the production of L19-AuNP conjugates.

### 2.3. Gold Nanoparticles Characterization

Both bare (AuNPbare) and L19-functionalized gold nanoparticles (L19-AuNP) were kindly provided by the company Nano Flow (Ougree, Belgium). NPs were characterized by dynamic light scattering (DLS) to register their hydrodynamic diameter (HD), polydispersity index (PDI), and zeta potential (ζ-potential). Tests were performed on fresh NPs (immediately after their production, t_0_) and again on aged NPs, after at least 17 months (t_17_) of storage in the dark at room temperature or at 4 °C, considering bare and L19-AuNPs, respectively. The two types of AuNPs were stored in phosphate-buffered saline (PBS), Tween 20 (0.05%, *w*/*v*), at the proper temperature, following the instructions provided by the company. A period of 17 months was considered, taking into account the expiry date of the different formulations suggested by the company. For the analyses, NPs were properly diluted in deionized water (1:50, *v*/*v*) and a particle size analyzer Zeta Sizer Nano ZS90 (Malvern Instruments Ltd., Worcestershire, UK) was used.

### 2.4. ELISA Validation

An ELISA was performed to screen the antibody clones, to confirm the binding activity of the purified L19 antibody, and to evaluate the binding activity of the gold nanoparticles decorated or not, with an antibody recognizing FN-EDB (L19-AuNP and AuNPbare, respectively). For all assays, 96-well black plates (BRAND) were coated overnight, at 4 °C, with 5 μg/mL of a fibronectin recombinant fragment constituted by type III repeats (positive antigen 7.B.8.9 or negative antigen FN7.8.9.10, the FN without EDB, SIRIUS Biotech s.r.l., Sunnyvale, CA, USA) and 2% bovine serum albumin (BSA, Fisher Scientific, Waltham, MA, USA) diluted in PBS. After washing with PBS containing 0.05% Tween 20, the wells were blocked for 2 h at room temperature (RT) with 2% BSA in PBS. Then, L19 samples (50 μL/well) were incubated for 2 h at RT, followed by incubation with a rabbit anti-uteroglobin (SIRIUS Biotech s.r.l.) (40 μg/mL) for 1 h, and an Alexa Fluor 594-conjugated anti-rabbit antibody (1:400, Invitrogen) for binding detection. To validate the binding activity, both gold nanoparticles (AuNPs) (50 μL/well) were incubated for 2 h at RT. After the final washes in PBS-Tween, fluorescence intensity (excitation wavelength 580 nm and emission wavelength 635 nm) and absorbance (525 nm and 540 nm) were measured using a plate reader (TECAN).

### 2.5. Immunohistochemistry Studies

Frozen sections of NB tumors (6 μm thick) were air-dried for 2 h and fixed in cold acetone for 10 min (min); endogenous peroxidase activity was quenched with 3.7% hydrogen peroxide in PBS for 40 min and non-specific sites were saturated with 1.25% normal goat serum (NGS) in PBS for 30 min.

To detect FN-EDB, sections were incubated with 5 microg/mL biotinylated L-19 antibody (4 °C, overnight), followed by streptavidin–peroxidase (Dako, Glostrup, Denmark) and ImmPACT^®^ DAB Substrate Kit (Vector Laboratories, Inc., Newark, CA, USA) as chromogen; sections were then counterstained with hematoxylin.

Negative controls were added to each session by omitting the primary antibodies and substituting them with NGS 1.25%. IHC staining was evaluated on images acquired with the Digital Pathology Slide Scanner Aperio AT2 DX System, Leica.

For histological characterization, the antibody L-19 was biotinylated using the EZ-Link^TM^ Sulfo-NHS-LC biotinylation kit (Thermo Fisher Scientific, Rockford, IL, USA cat # 21435) according to the manufacturer’s instructions and validated following the HABA assay.

The L19 conjugation with Alexa 488 was performed following the manufacturer’s instructions reported for the commercial kit Alexa Fluor™ 488 Protein Labeling Kit, cat.# A10235, Invitrogen Molecular Probes, Eugene, OR, USA.

### 2.6. Photoacoustic Imaging

In vitro phantom analysis: to allow detection of L19-AuNP in vivo, we previously characterized their absorption spectra in vitro using Spectro Mode, across the whole wavelength range of 680–970 nm, and the Vevo Contrast PHANTOM chamber. After considering oxy and deoxy-hemoglobin interference, the optimal wavelengths were identified at 684 and 708 nm and set for unmixing the signal in vivo (Vevo LAZR Software Option 50025–Multiplexer, FUJIFILM VisualSonics, Inc., Toronto, ON, Canada).

In vivo imaging: Anatomic and molecular imaging were carried out on two PDX mouse models and two tumor-free mice, respectively (Figure 1). A high-frequency ultrasound transducer MX400 (30 MHz center frequency), which enables a special resolution of 5 μm and imaging depth of 5–6 cm, combined with optical fibers for laser application (FUJIFILM VisualSonics, Inc.), was used. Photoacoustic (PA) imaging was conducted on two PDX tumor-bearing animals with an average volume of 0.8 cm^3^. Two tumor-free nu/nu mice were used as controls. Mice were kept under sedation (induction with 3–4% isoflurane; maintenance with 1.5% isoflurane) and placed on the instrument’s thermal platform to maintain the body temperature at 37 °C and continuous recording of vital parameters (heart and respiration rates, body T °C) during the entirety of imaging acquisition (45 min). During analysis, animals were maintained in a prone position for imaging the tumor, spleen, and kidneys, then changed to a supine position to visualize the liver. Bubble-free ultrasound imaging gel was placed on the skin, and axial sections were acquired in PA mode in the 680–980 wvl range. Since *nu*/*nu* mice are albino, the images were acquired with no interference from melanin. The PA gain was set at 40 dB. Tumor and kidneys were acquired in 3D, with a step size of 0.4 mm within a range of 9.5 mm, using a motorized Vevo Rail system (FUJIFILM VisualSonics, Inc.).

All animals (PDX pt#1, PDX ptx#2, and 2 tumor-free mice) were first analyzed to detect basal signals, then injected intravenously with 100 μL of L19-AuNP (final optical density of 24.3) for post-injection acquisition (Figure 1). Three time points at 6, 9, and 24 h post-injection were selected as most representative of the rapid surge and decline in the PA signal and according to previous studies, indicating a different retention time in target and non-target organs on the basis of AuNP dimensions, morphology, and coating [33,34,35,36]. PA signals were then calculated with the multiplexer software (FUJIFILM VisualSonics, Inc.), drawing a Region of Interest (ROI) in each image and considering the “PA average threshold” parameter, in “Unmixing mode”, during post-processing analysis (Vevo LAB V.3.2.5. software, FUJIFILM VisualSonics, Inc.). Values were reported as differences in signal levels (DPA) between each post-injection time point (PA_tx_) and basal level (PA _pre inj_), according to the formula DPA = PA_tx_ − PA _pre inj_.

### 2.7. Statistical Analysis

All in vitro experiments were performed at least three times. The analyses were performed using GraphPad Prism version 5.00 for Windows (GraphPad, La Jolla, CA, USA). One-way analyses of variance (ANOVA) with Tukey’s Multiple Comparison Test were used to evaluate differences in the ELISA.

## 3. Results

### 3.1. FN-EDB Expression in Mouse Models of Neuroblastoma

**Figure 2:**
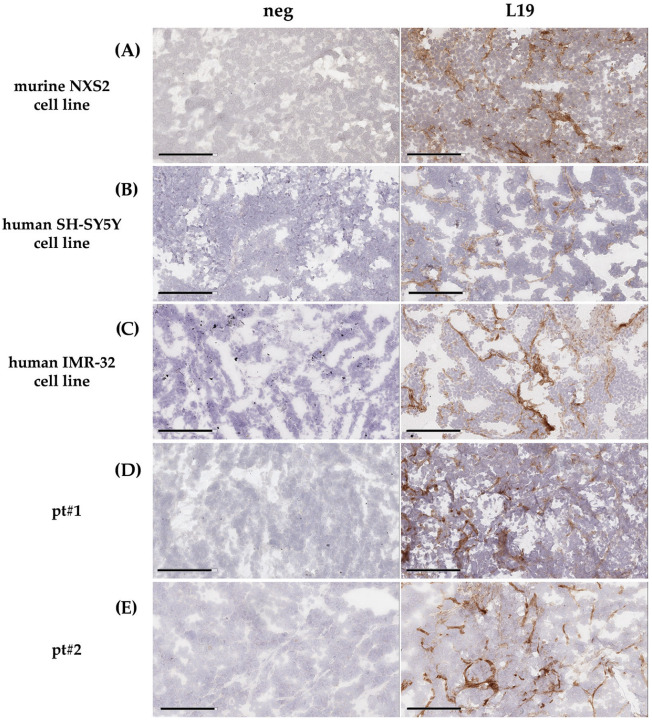
**Immunohistochemical analysis of FN-EDB expression in animal models of neuroblastoma**. Representative images of neuroblastoma tumors, obtained by the inoculation of (**A**) murine (NXS2) and (**B**–**E**) human (SH-SY5Y and IMR-32) cells in the left adrenal gland of mice (**B**,**C**) or by subcutaneously xenografting patient-derived tumor fragments (PDX) of neuroblastoma patient 1 (pt#1) and patient 2 (pt#2) (**D**,**E**), stained with anti-FN-EDB (L19) antibody. neg: Negative control, obtained by omitting biotinylated L19 primary antibody. Bar: 200 μm.

FN-EDB expression was evaluated by immunohistochemical (IHC) analysis in both cell line-derived and patient-derived animal models of NB. Mouse NXS2 cell line-derived tumors display a strong positivity for FN-EDB (Figure 2A), confirming previous results obtained in a syngeneic NB model [37]. In a subsequent experiment, a more clinically relevant animal model of NB was used. *MYCN* single-copy (SH-SY5Y) and *MYCN*-amplified (IMR-32) human NB cell lines were orthotopically injected in mice. Again, these cell line-derived tumors exhibited strong positivity for FN-EDB (Figure 2B,C). As expected, the immunoreactive signal was mainly localized in the tumor vasculature and in the cortical area, delineating a matrix deposition surrounding tumor cells (Figure 2A–C).

Finally, in patient-derived animal models of NB (PDX), tumors displayed a pattern of FN-EDB distribution similar to that seen in cell line-derived models, outlining cell aggregates typical of regions with blood vessel formation (Figure 2D,E).

Altogether, the results obtained in multiple NB models suggest a potential role of FN-EDB in tumor targeting by the use of FN-EDB-directed nanoparticles.

### 3.2. L19-AuNP Characterization

Both bare (AuNPbare) and L19-functionalized gold nanoparticles (L19-AuNP) were characterized in terms of size, polydispersity, and zeta potential, pivotal for their clinical translation. These chemical–physical parameters were also considered after several months of storage. As reported in Table 1, both formulations evaluated after production (t_0_) were sufficiently small in size and homogenously negatively charged. As expected [38], an increase in diameter and zeta potential between L19-AuNP and AuNPbare demonstrated that the surface modification with L19 occurred correctly on AuNP.

Interestingly, L19-AuNP evaluated after months of storage at 4 °C demonstrated excellent stability over time, unlike AuNPbare, which has shown an important increase in PDI and zeta potential (Table 1), making them unsuitable for use in in vivo studies.

### 3.3. In Vitro Binding Specificity of Gold Nanoparticles Decorated with L19 Antibody Recognizing FN-EDB

**Figure 3:**
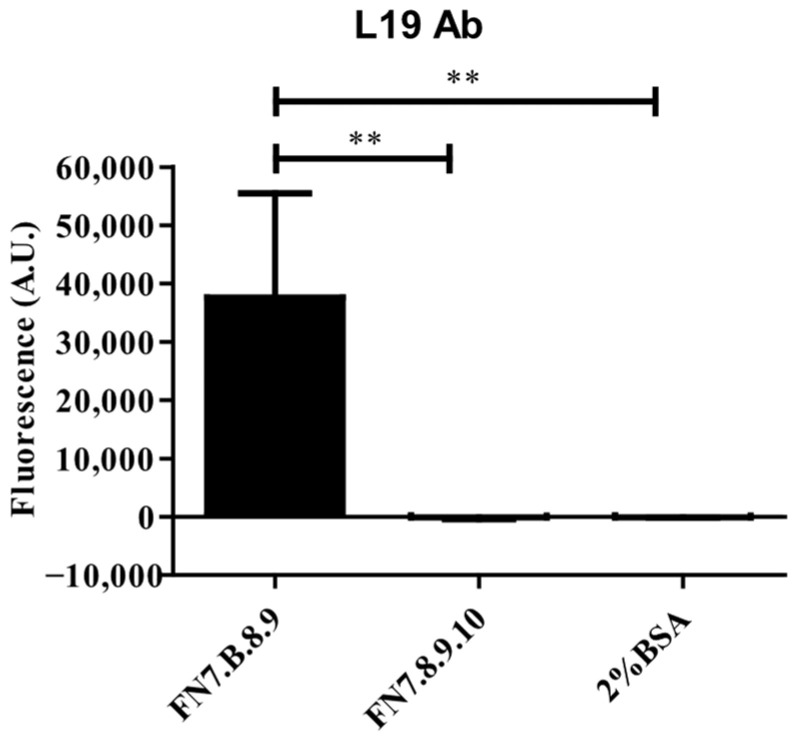
**Binding specificity of free L19 antibody to FN-EDB detected by ELISA.** FN7.B.8.9: Positive antigen; FN7.8.9.10: negative antigen (FN without EDB); 2%BSA: 2% bovine serum albumin in PBS, negative control (blocking agent). Statistics: **, *p* < 0.01.

The binding activity of the purified L19 antibody, both free and coupled to gold nanoparticles (AuNPs), was tested by ELISA. First, the results obtained by using L19 antibody (Ab) indicated its strong and specific binding activity to the target FN-EDB (Figure 3). Indeed, the fluorescence signal recorded on FN7.B.8.9-coated wells was significantly higher than those measured in the wells coated with both FN7.8.9.10 and 2%BSA negative controls, where negligible fluorescence values were detected (Figure 3).

**Figure 4:**
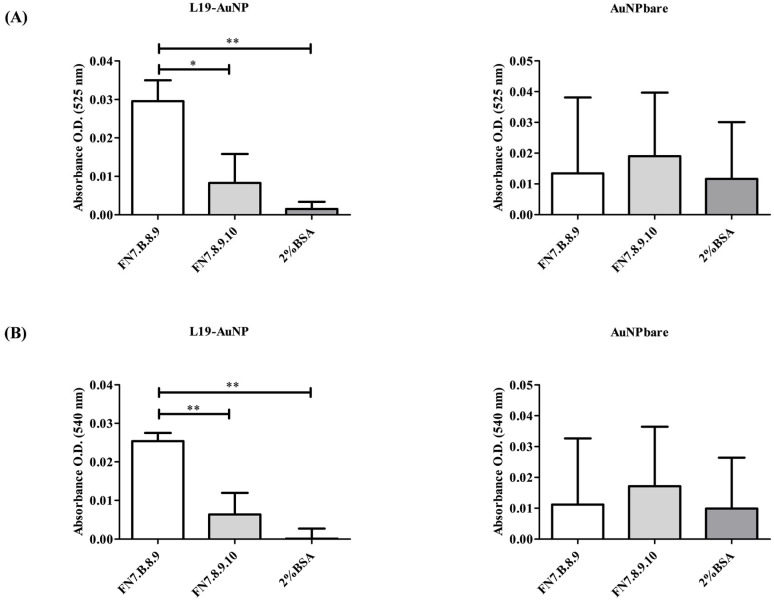
**Binding specificity of L19-functionalized gold nanoparticles to FN-EDB.** Binding of L19-functionalized gold nanoparticles (L19-AuNP) and non-functionalized gold nanoparticles (AuNPbare) detected by ELISA at 525 nm (**A**) and 540 nm (**B**). FN7.B.8.9: Positive antigen; FN7.8.9.10: negative antigen (FN without EDB); 2%BSA: 2% bovine serum albumin in PBS, negative control (blocking agent). Statistics: *, *p* < 0.05; **, *p* < 0.01.

Subsequently, L19 Ab was coupled at the external surface of AuNPs, and the binding activity of L19-AuNP was evaluated by ELISA at 525 nm and 540 nm absorbance. As shown in Figure 4, L19-AuNP showed specific binding to FN7.B.8.9-coated wells, resulting in significantly enhanced binding when compared to that obtained on both FN7.8.9.10 and 2%BSA negative controls at both wavelengths used. On the contrary, non-functionalized nanoparticles (AuNPbare) showed unspecific binding to all coated supports used, indicating that the conjugation of L19 Ab to AuNPs preserved its selective recognition of FN-EDB (Figure 4).

### 3.4. In Vivo L19-AuNP Tumor Accumulation

In the in vivo experiments, to evaluate the tumor-targeting ability of FN-EDB-recognizing NP, a time course study at baseline (immediately before injection) and at 6, 9, and 24 h post L19-AuNP intravenous injection was performed in the two FN-EDB-expressing (Figure 1) PDX models of NB.

Due to their poor stability (Table 1), and above all, insufficient binding specificity to FN-EDB in vitro (Figure 3), AuNPbare has not been tested in vivo.

**Figure 5:**
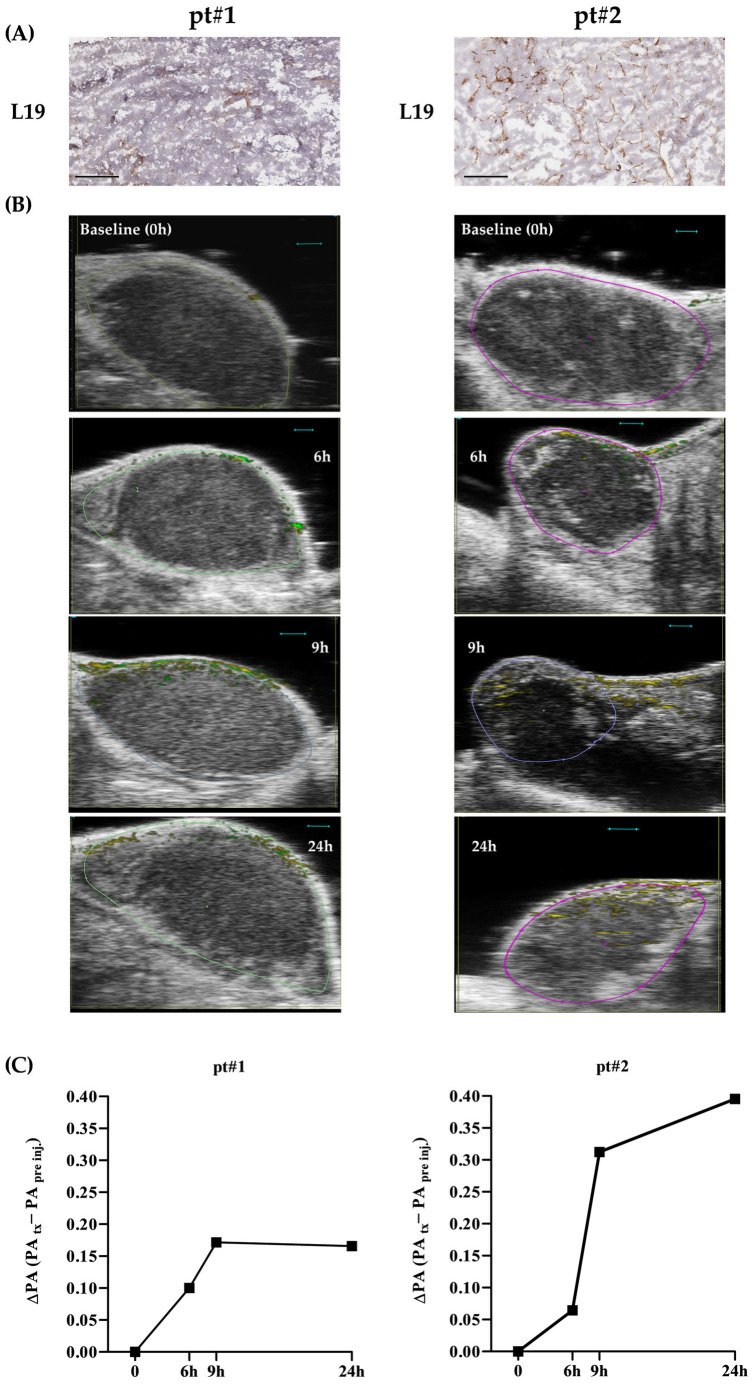
**Tumor homing of L19-AuNP in patient-derived xenografts** (**PDX**) **of neuroblastoma evaluated with photoacoustic imaging.** (**A**) Immunohistochemical staining of FN-EDB in PDX derived from neuroblastoma patient #1 (pt#1) and patient #2 (pt#2). Bar: 300 μm. (**B**) Representative photoacoustic images of pt#1 and pt#2 tumors, at baseline and 24 h after L19-AuNP injection. Blue bar: 1 mm. green and yellow dots indicate the locations of PA signals acquired at two distinct wavelengths, 684 and 708 nm, respectively; the tumor area is outlined by a solid line. (**C**) Time-dependent accumulation of L19-AuNP in the tumor in the two PDX models (pt#1 and pt#2) at baseline and 6, 9, and 24 h after L19-AuNP injection; at least 50 images per tumor were analyzed, and the reported value represents their mean.

The NP’s fate was first assessed by recording the time-dependent photoacoustic (PA) signal of L19-AuNPs detected in the tumor. Images were acquired in 3D mode throughout the whole length of the tumor along the head-to-tail axis. As shown in Figure 5, tumor uptake of L19-AuNPs was time-dependent. Specifically, in pt#1, which displayed an FN-EDB expression mainly in the marginal part of the tumor (Figure 5A), L19-AuNPs reached a peak of accumulation at 9 h post-injection, remaining stable up to 24 h (Figure 5B,C). In pt#2, having a wider distribution of FN-EDB throughout the tissue specimen (Figure 5A), the accumulation of L19-AuNPs was much higher compared to that observed in pt#1 at all time points analyzed, reaching a peak after 24 h post-injection (Figure 5B,C). The results obtained suggest a specific correlation between increased FN-EDB expression and enhanced tumor homing (Figure 5).

**Figure 6:**
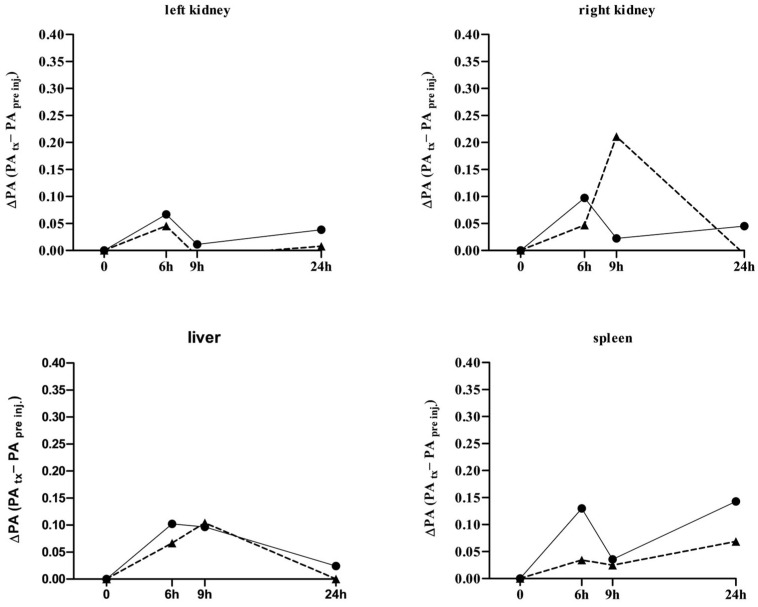
**Biodistribution study of L19-AuNP in tumor-free mice evaluated with photoacoustic imaging.** A preliminary time-dependent L19-AuNP biodistribution study was performed in two mice, identified with a black circle and a black triangle, respectively, at the basal condition (0) and 6, 9, and 24 h post L19-AuNP injection. At least 30 images per organ were analyzed, and the reported values represent their mean.

The biodistribution (BD) profile of L19-AuNP was evaluated in the kidneys, liver, and spleen. L19-AuNP accumulation was negligible and almost disappeared 24 h after administration (Figure 6).

## 4. Discussion

In this study, we demonstrate that the fibronectin extra-domain B (FN-EDB) is expressed in the tumor microenvironment of neuroblastoma (NB). We also show that a gold nanoparticle (AuNP), functionalized with the L19 antibody, is able to specifically recognize FN-EDB (L19-AuNP) and specifically home into patient-derived xenograft (PDX) models of NB, suggesting the possible role of FN-EDB as a novel target molecule in the clinical setting for NB patients.

The expression of FN-EDB was demonstrated, ex vivo, in NB models obtained both by orthotopically injecting NB cell lines in the adrenal gland [10] and by implanting NB tumor patient-derived xenografts stored in our repository [32]. The results obtained indicate that FN-EDB is a marker consistently expressed in NB, albeit at varying levels, which may depend on tumor development, aggressiveness, and neovascularization. Our findings are in line with previous studies reporting FN-EDB expression as a common feature across different tumor types [8], including NB [37]. FN-EDB expression correlates with microenvironment features associated with increased tumor malignancy, such as fibrosis, neoangiogenesis, and metastatic invasiveness [39].

In this study, we employed the L19 scFv fused to uteroglobin. Although antibody affinity for the target is a key determinant of tumor targeting [29], other parameters—such as molecular size, format stability, and avidity—also play crucial roles in shaping pharmacokinetics and in vivo performance. To systematically investigate these factors, various fully human formats of the L19 antibody have been compared in previous studies [30,40]. Among these, we selected the uteroglobin-based format due to its exceptional biochemical stability. Uteroglobin is a small human homodimeric protein (15.8 kDa per monomer) characterized by high resistance to pH and temperature variations, protection from proteolytic degradation, and remarkable solubility [41]. Its covalent dimeric structure, stabilized by disulfide bridges, provides a compact and robust scaffold, while a central hydrophobic cavity is capable of accommodating small hydrophobic molecules. Owing to these features, uteroglobin has been widely used as a core scaffold for the generation of polyvalent and multispecific protein constructs. The resulting L19-uteroglobin construct is a bivalent molecule of approximately 80 kDa, consisting of two L19 scFv fragments linked through the uteroglobin dimer. This format displays excellent solubility (>2 mg/mL in PBS) and high stability and can be lyophilized and reconstituted without a loss of activity or aggregation. Moreover, L19-uteroglobin fusion proteins can be efficiently produced in *E. coli* [42], enabling cost-effective and scalable manufacturing.

Owing to its strong tumor–stroma specificity and negligible reactivity in healthy tissues, L19 Ab has become a widely used targeting ligand in oncology for delivering imaging agents and therapeutic cargos directly to the tumor microenvironment. Its stability, small size, and preserved binding performance after chemical conjugation make L19 Ab particularly suitable for the surface engineering of nanosystems. Among the different nanosystems, AuNPs, due to their versatility, could be easily engineered with antibodies, making them specific for antigen recognition. In this work, the studied AuNPs were successfully conjugated with L19 and tested for their clinical potential in NB by using photoacoustics (PA imaging). Here, the combined results obtained from the analysis of both free L19 Ab and L19-AuNPs confirm the preservation of binding specificity, even after its conjugation, toward the FN-EDB. Indeed, in all ELISA assays performed, wells coated with the FN-EDB substrate FN7.B.8.9 consistently displayed significantly higher fluorescence or absorbance values compared to negative controls. The strong signal reproducibility between independent production batches of L19 Ab highlights the robustness of the Ab production and purification protocol and indicates that the structural integrity of L19 was not affected during production or subsequent conjugation onto the nanoparticle surface. Indeed, the results obtained demonstrate that the immobilization of L19 Ab on the AuNP surface does not impair its functional recognition of the target FN7.B.8.9, validating the functional stability of the conjugate for further biological and in vivo applications. In contrast, consistent with the intrinsic “sticky” surface behavior of unfunctionalized AuNP, AuNPs lacking the L19 Ab coating (AuNPbare) displayed variable, non-specific interactions with either positive or negative control substrates. However, despite this intrinsic non-specificity, the behavior of AuNPbare represented an essential tool in vitro to demonstrate that the selective binding observed with L19-AuNP was exclusively attributable to the antibody’s presence and activity rather than to non-specific NP–protein interactions. In conclusion, the lack of AuNP binding specificity, together with their unstable chemical–physical features, renders them unsuitable for further in vivo investigations.

Recently, PA has emerged as a novel, non-invasive molecular imaging technique that combines the advantages of both optical and ultrasound modalities [43]. PA imaging offers higher optical resolution than fluorescence imaging and better tissue contrast than ultrasound. Notably, the absence of ionizing radiation also makes PA imaging safer than computed tomography and radionuclide-based techniques [23]. In addition, PA provides real-time information with spatial resolutions down to 5 μm and imaging depths of up to 5–6 cm. Although in our study, PA imaging was performed in subcutaneous PDX models of NB, these resolution capabilities make L19-AuNP-assisted PA imaging suitable for evaluating internal tumors as well, such as orthotopic NB tumors growing in the adrenal gland [44].

AuNPs are among the most promising PA contrast agents for both diagnosis and therapy [23,25,26], owing to their safety profile [25,26] and their favorable optical properties. When excited by laser energy, the size and shape of AuNPs undergo changes that alter their resonant frequency. This phenomenon, known as surface plasmon resonance, enables the use of wavelengths within the ‘biological window’ (650–1100 nm), where blood and tissue attenuation are minimal, thus enhancing the contrast generated by these particles [44]. In our work, to compensate for shifts in the L19-AuNP absorption spectra [44]—also influenced by varying degrees of accumulation in FN-EDB-rich sites—measurements were acquired at two distinct peaks, 684 and 708 nm. Since we used athymic *(nu*/*nu)* mice, no interference from melanin or hair occurred; however, when translating our experimental settings to other models, a careful preliminary assessment of potential endogenous sources of interference is required.

When tested in two subcutaneous PDX models of NB, we demonstrated, in vivo, that L19-AuNP rapidly accumulates in tumors within 24 h after injection. Moreover, we observed that the post-injection PA signal intensity of L19-AuNP specifically correlated with the amount of FN-EDB expression in the corresponding tumor masses, validating the PA imaging system in our experimental setup. In contrast, the signal levels of L19-AuNP in non-target organs were much lower, suggesting a good clearance rate, consistent with reports on the safety of AuNPs as in vivo nanotracers/nanocarriers [26,45,46]. The results obtained also support the over-time use of L19-AuNP-assisted PA imaging as a minimally invasive tool in preclinical models. Indeed, this would enable repeated tumor mass measurement and FN-EDB content analysis, providing a novel parameter that might improve NB staging and progression assessment and extending the use of PA imaging in preclinical oncology research [24] for both diagnostic and therapeutic purposes. For example, it could become a tool to counteract neoangiogenesis progression. Indeed, while neoangiogenesis is a key process driving tumor development and metastasis, therapeutic approaches targeting its progression, such as anti-VEGF inhibitors, raise some concerns for their systemic vascular complications [47,48]. Therefore, having a local target could help minimize side effects and might open the door for a novel management strategy for NB patients.

In this study, L19-AuNPs were stored over time at 4 °C, while unfunctionalized AuNPbare was stored at room temperature, following the storage protocols provided by the manufacturer. All the AuNPs used, evaluated after production, were sufficiently small in size and homogenously negatively charged. To note, different temperatures of storage could affect the AuNP size, as previously reported [13,14]; high temperatures provide, indeed, kinetic energy sufficient to allow NP aggregation.

In this study, L19-AuNPs were stored at 4 °C and remained stable in terms of size and polydispersity index (PDI). The increase in the negative zeta potential of L19-AuNPs after 17 months can instead be explained by the long-term stabilization dynamics of the antibody–nanoparticle interface. Over extended storage, two concurrent processes may occur: reorientation of the surface-bound antibodies and/or increased surface accessibility of ionic groups. In the first case, antibodies can gradually rearrange on the nanoparticle surface, exposing a larger fraction of their negatively charged domains. This slow conformational relaxation has been documented in antibody–nanoparticle conjugates and leads to a more negative zeta potential over time [49,50,51]. In the second case, the aging of the hydration layer may increase the exposure of the charged residues at the nanoparticle interface, resulting in a more negative electrostatic profile without affecting particle integrity. Importantly, no aggregation or changes in hydrodynamic diameter were observed, confirming that the conjugates remained colloidally stable and structurally intact. AuNPbare was stored at room temperature, showing a significant increase in PDI (*p* = 0.0008) and zeta potential (*p* = 0.0002). These enhanced parameters, evaluated 17 months after their production, were probably due to the presence of nanoparticle aggregates, and for this reason, they were not tested in the tumor homing and biodistribution studies. To note, both AuNPs were stored in phosphate-buffered saline (PBS), Tween 20 (0.05%, *v*/*v*), and bovine serum albumin (1%, *w*/*v*) at the proper temperature, following the instructions provided by the company. Although the long-term measurements of hydrodynamic diameter, PDI, and zeta potential provided an initial indication of the colloidal behavior over storage, a comprehensive assessment of reproducibility and stability under biologically relevant environments needs to be further evaluated.

The specific uptake of L19-AuNP was demonstrated in only two PDX models of NB and needs to be undoubtedly confirmed in several samples from NB patients. However, although preliminary, this study demonstrates that FN-EDB is expressed in the tumor microenvironment of NB models developed from both *MYCN* single-copy and *MYCN*-amplified tumors, with a slight prevalence of expression in the amplified ones, suggesting FN-EDB expression as a potential new biomarker in the stratification of patients affected by NB. Furthermore, this study demonstrates that FN-EDB is responsible for the homing of the L19-functionalized AuNP into the tumor and that such selective targeting increases as a consequence of increased EDB expression in the tumor.

A further limitation of this work is the fact that a time-dependent study of the expression of FN-EDB was not performed. Indeed, an aspect that would be interesting to analyze could be represented by the study of the expression of EDB in the NB tumor at the onset, in relapse, or during standard-of-care treatments in order to validate this receptor as a constitutive and stable marker or as a marker expressed only in particular phases of the disease.

NB, particularly in refractory or relapsed cases, remains a significant clinical challenge in pediatric oncology. This study is based on the hypothesis that the development, in the future, of gold nanoparticles co-loaded with standard of care for NB, and functionalized with L19 antibody, might enhance diagnostic, follow-up, and therapeutic specificity for NB. By leveraging the high imaging sensitivity of AuNP for PA, the platform proposed could offer real-time, non-invasive biodistribution and tumor homing of the proposed nanosystem, without the use of radioactive agents currently used in clinic [52], while delivering antitumor compound selectivity to the tumor site.

## Figures and Tables

**Table 1:** **Z-average particle size** (**hydrodynamic diameter**), **polydispersity index, and zeta potential of gold nanoparticles.** Gold nanoparticles, L19-functionalized (L19-AuNP) or untargeted (Au-NPbare), were evaluated for their hydrodynamic diameter (HD), polydispersity index (PDI), and zeta potential after their production (t_0_) or after at least 17 months of storage (t_17_). Results are expressed as the average values ± standard deviation.

	HD (nm)	PDI	ζ-Potential (mV)
**L19-AuNP** (**t_0_**)	87.21 ± 13.66	0.240 ± 0.030	−27.17 ± 1.16
**L19-AuNP** (**t_17_**)	105.23 ± 2.08	0.296 ± 0.035	−39.28 ± 0.99
**AuNPbare** (**t_0_**)	55.87 ± 6.72	0.220 ± 0.070	−38.37 ± 2.33
**AuNPbare** (**t_17_**)	59.69 ± 3.35	0.472 ± 0.035	−51.18 ± 0.49

## Data Availability

The raw data supporting the conclusions of this article will be made available by the authors on request.
